# Supplementary material for: Classification aware neural topic model for COVID-19 disinformation categorisation
Source: PLoS One. 2021 Feb 18;16(2):e0247086. doi: 10.1371/journal.pone.0247086 (PMC7891716; doi:10.1371/journal.pone.0247086)
Supplement: S1 Appendix — (PDF) [file pone.0247086.s001.pdf]

S1 Appendix - Data Structure and Example IFCN Web Page

S1 Fig. The screen shot of IFCN debunk page. Information enclosed in the boxes is extracted into the respective data fields of our dataset.

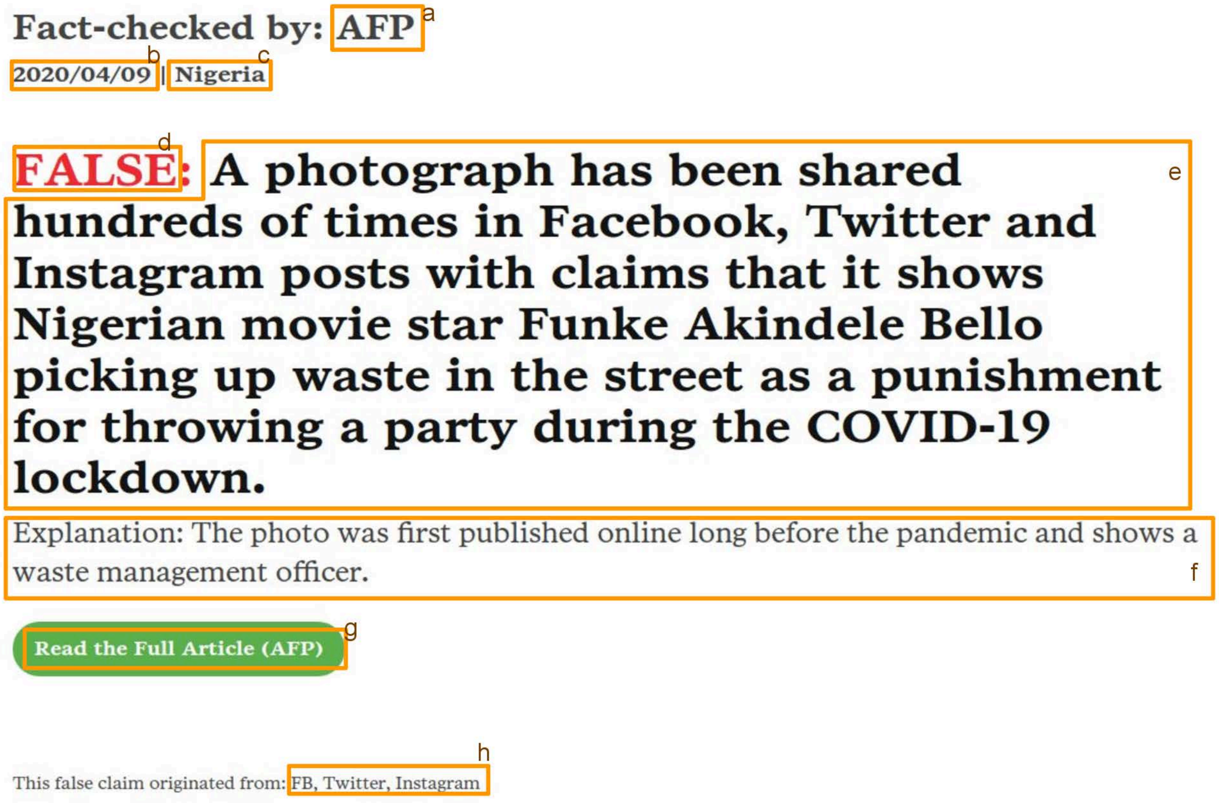

S1 Table. COVID-19 disinformation category data structure

Label Fields	Extraction Method	Example
a. Debunk Date	IFCN HTML	2020/04/09
b. Claim	IFCN HTML	A photograph ... lockdown.
c. Explanation	IFCN HTML	The photo was ... officer.
d. Source link	IFCN HTML	factcheck.afp.com/photo-was...
e. Veracity	String Match	False
f. Originating platform	String Match	Facebook, Twitter, Instagram
g. Source page language	langdetect	English
h. Media Types	JAPE Rule	Image
i. **Categories**	Manually annotated	Prominent actors

The description of these fields is as follows:

(a) 'Debunk Date': The date of publication of this debunk on the IFCN Poynter website (IFCN HTML).
(b) 'Claim': The false claim and its rephrasing/summary by the fact-checker (IFCN HTML).
(c) 'Explanation': The explanation of why this is a false claim, as provided by the fact checkers (IFCN HTML).
(d) 'Source link': The link to the original page of the debunk, as published on the fact-checking organisation's website (IFCN HTML).
(e) 'Veracity': This label is extracted from the IFCN HTML tags and post-processed by string matching (for full details please refer to S2 Appendix B). The value is one of:
    - **False** – The claim of the information is totally false;
    - **Partially False** – The information is a mix of true and false;
    - **Misleading** – The claim of the information is true but leads in a wrong direction;
    - **No evidence** – No evidence to prove the information is correct or not.
(f) 'Originating platform': The platform where the disinformation spread originally, e.g. Facebook, news, etc. This label is extracted from IFCN HTML tags and post-processed by string matching.
(g) 'Source page language': The main language used in the source page. The language is detected using the langdetect Python package[22] applied to the debunk text.
(h) 'Media type': The main media type of the disinformation, i.e. image, video, text and audio. We apply a JAPE rule-based extractor over 'Claim', 'Explanation', 'Claim Origin' and the debunk text to extract the media type information. The motivation for this rule-based extraction in given in S3 Appendix C.
(i) 'Category': The 10 COVID-19 disinformation categories based on [3]: Public authority; Community spread and impact; Medical advice, self-treatments, and virus effects; Prominent actors; Conspiracies; Virus transmission; Virus origins and properties; Public Reaction; Vaccines, medical treatments, and tests; and Other. Please refer to S4 Appendix D for the full description of these categories. This field was partially labelled by human annotators (the full process is described in Section 2). The categories for the remaining unlabelled data were assigned using CANTM (described in Section 3).

[22] `https://pypi.org/project/langdetect/`
