# Supplementary material for: Classification aware neural topic model for COVID-19 disinformation categorisation
Source: PLoS One. 2021 Feb 18;16(2):e0247086. doi: 10.1371/journal.pone.0247086 (PMC7891716; doi:10.1371/journal.pone.0247086)
Supplement: S2 Appendix — (PDF) [file pone.0247086.s002.pdf]

S2 Appendix – The String Matching Process

Values in the IFCN data fields are manually provided by the fact-checkers. Therefore, the raw text may contain typos (e.g. Facebook as Facebok), acronyms (e.g. Facebook as FB) and different terminology or expressions (e.g. Partially False vs Partially True in the Label field). In order to streamline the data, a string matching process is applied to normalise the data in the d. Veracity and h. Originating platform fields.

The String Matching process maps free text into standard unified values defined in this work. The pseudo-code of the cleaning process is illustrated in S Algorithm 1. We created string-mapping lists for each field by manually inspecting text values in the respective field. Each list contains pairs of (standard value, raw word list). The standard value is assigned when the text contains words from the raw word list. An example of a mapping list is shown in S2 Table, where the first column contains the standard values, and the second column are the lists of raw words. For example, the Facebook acronym ‘FB’ or typo ‘faceboos’ in the raw text will map to the standard value “Facebook”. The full matching list is shared with the source code

S Algorithm 1: String Matching Process

```
Input   : ClaimOrigin, Label
Output  : cleanedClaimOrigin, cleanedLabels
Function valueMapping(text, mappingList):
    organisedValues = [];
    for words in text do
        for StandardValue in mappingList do
            if word in mappingList[StandardValue] then
                organisedValues.append(StandardValue)
        end
    end
    return organisedValues;
end
cleanedLabels = valueMapping(Label, LabelMappingList);
cleanedClaimOrigin = valueMapping(ClaimOrigin, LabelMappingList);
```

**Standard value**	**Raw word list**
Claim Origin Mapping List	
Facebook	FB, faceboos, facebook,facebok ...
YouTube	youtube, youtuber
...	...
Label Mapping List	
False	Pants on Fire!, False, Fake news, Incorrect, ...
Partially False	Partially correct, mostly false, half truth, ...
...	...
Media Type Mapping List	
Image	photo, photograph, image, picture, ...
Audio	radio, audio
...	...

S2 Table. Example String Matching list
