# Supplementary material for: Classification aware neural topic model for COVID-19 disinformation categorisation
Source: PLoS One. 2021 Feb 18;16(2):e0247086. doi: 10.1371/journal.pone.0247086 (PMC7891716; doi:10.1371/journal.pone.0247086)
Supplement: S3 Appendix — (PDF) [file pone.0247086.s003.pdf]

S3 Appendix - Rule-Based Extraction of Media Type

The main modality of debunked disinformation can be one of four media types: Text, Image, Video and Audio. Sometimes disinformation may consist of several media types, e.g. an image and accompanying textual narrative. The main media type of a given disinformation instance is considered the one highlighted by the fact-checker as the main carrier of the false content. For example, for the disinformation in S1 Fig, image (photograph) is the media type making people believe that Funke Akindel Bello was punished.

S Algorithm 2 shows the pseudo code for media type enrichment. In general, the media type can be extracted based on:

1. the originating platform: for some platforms, the media type can be derived unambiguously. For example, if the originating platform is TV, the media type must be a video.

2. Claim and Explanation of the disinformation: In the IFCN debunks, the main media type is normally described in the Claim and/or Explanation by the fact-checkers. For example, from the text of the Claim shown in S1 Fig 'A photograph has been shared ...'), it can be deduced that the media type is Image. Descriptions of media types can easily be extracted using the same string matching approach described above.

3. the text of the debunk page, extracted from the source URL: This text is much longer and noisier than the short, fact-checker authored IFCN Claim/Explanation fields. Therefore, in order to capture all these cases we implemented JAPE [74] rule-based mappings instead of simple string or regular expression matching. JAPE is a pattern-based NLP engine [75], which provides better generalisation and accuracy then simple word matching. All JAPE rules are shared in the source code.

S Algorithm 2: Media Type Extraction

```
Input  : Claim Origin, Claim, Explanation, SourcePageEnglish
Output : MediaType
MediaTypeInClaim = valueMapping(Claim);
MediaTypeInExplaination = valueMapping(Explanation);
MediaTypeInSoucePage = JAPErule(SourcePageEnglish);
if (Claim Origin == YouTube) or (Claim Origin == TV) then
  | MediaType = Video;
else if (MediaTypeInClaim != None) then
  | MediaType = MediaTypeInClaim;
else if (MediaTypeInExplaination != None) then
  | MediaType = MediaTypeInExplaination;
else if (MeduaTypeInSoucePage != None) then
  | MediaType = MeduaTypeInSoucePage;
else
  | MediaType = None
end
```
