# Supplementary material for: Classification aware neural topic model for COVID-19 disinformation categorisation
Source: PLoS One. 2021 Feb 18;16(2):e0247086. doi: 10.1371/journal.pone.0247086 (PMC7891716; doi:10.1371/journal.pone.0247086)
Supplement: S4 Appendix — (PDF) [file pone.0247086.s004.pdf]

S4 Appendix - Definitions of the COVID-19 Disinformation Categories

- Public authority: Claims about policy, action, or communication by a public authority (e.g. government department, police, fire brigade, government officials), including claims about WHO guidelines and recommendations as well as those about governments' action or advice.
- Community spread and impact: Claims about people, groups, or individuals with regard to how the virus is spreading (internationally, regionally, or within more specific communities); impact on people, groups (including religions and ethnic minorities), or individuals; deaths, etc.
- Medical advice, self-treatments, and virus effects: Claims about health remedies, self-treatments, self-diagnosis, signs and symptoms, effects of the virus, etc.
- Prominent actors: Claims about pharmaceutical companies, media organisations, health-care supply businesses, other companies, or famous people (including celebrities and politicians). Note that this does not include claims made bypoliticians or other famous people unless they are about other prominent actors.
- Conspiracies: Claims that the virus was created as a bioweapon, that some organization supposedly created the pandemic, that it was predicted, etc.
- Virus transmission: Claims about how the virus is transmitted and how to prevent transmission. This includes cleaning as well as use of specific lighting, appliances, protective equipment, etc.
- Virus origins and properties: Claims about the origins of the virus (e.g,. in animals) or its properties.
- Public Reaction: Claims that encourage hoarding, buying supplies, practising or avoiding social distancing, compliance or non-compliance with public health measures, protests and civil disobedience against official measures (including government measures). etc.
- Vaccines, medical treatments, and tests: Claims about vaccines, tests, and treatments, including the development and availability of a vaccine or a treatment. (Claims about self-treatment fall under the medical advice category, however.)
- Other: Use this category if the claim does not fit into any category above, if it does not seem to contain misinformation, or if you cannot read the language or understand the text for any reason.
