# Supplementary material for: Classification aware neural topic model for COVID-19 disinformation categorisation
Source: PLoS One. 2021 Feb 18;16(2):e0247086. doi: 10.1371/journal.pone.0247086 (PMC7891716; doi:10.1371/journal.pone.0247086)
Supplement: S5 Appendix — (PDF) [file pone.0247086.s005.pdf]

S5 Appendix - Deriving the ELBO

This section describes the details of $ELBO_{x_{bow}}$ and $ELBO_{x_{bow,\hat{y}}}$ derivation and calculation.

$$
\begin{aligned}
& z \sim q(z|x) \\
& \log p(x_{bow}) = \mathbb{E}_z \log p(x_{bow}) \\
& = \mathbb{E}_z[\log p(x_{bow}, z)] - \mathbb{E}_z[\log p(z|x_{bow})] \\
& = \mathbb{E}_z[\log \frac{p(x_{bow}, z)}{q(z|x)}] - \mathbb{E}_z[\log \frac{p(z|x_{bow})}{q(z|x)}] \\
& = EBLO_{x_{bow}} - D_{KL}(p(z|x_{bow})||q(z|x)) \\
& = EBLO_{x_{bow}} + D_{KL}(q(z|x)||p(z|x_{bow}))
\end{aligned}
$$

$$
\begin{aligned}
& ELBO_{x_{bow}} \\
& = \mathbb{E}_z[\log p(x_{bow}, z)] - \mathbb{E}_z[\log q(z|x)] \\
& = \mathbb{E}_z[\log p(x_{bow}|z)] + \mathbb{E}_z[\log p(z)] - \mathbb{E}_z[\log q(z|x)] \\
& = \mathbb{E}_z[\log p(x_{bow}|z)] - D_{KL}(q(z|x)||p(z))
\end{aligned}
$$

$$
\begin{aligned}
& z_s \sim q(z|x, \hat{y}) \\
& \log p(x_{bow}, \hat{y}) = \mathbb{E}_{z_s} \log p(x_{bow}, \hat{y}) \\
& = \mathbb{E}_{z_s}[\log p(x_{bow}, \hat{y}, z_s)] - \mathbb{E}_{z_s}[\log p(z_s|x_{bow}, \hat{y})] \\
& = \mathbb{E}_{z_s}[\log \frac{p(x_{bow}, \hat{y}, z_s)}{q(z_s|x, \hat{y})}] - \mathbb{E}_{z_s}[\log \frac{p(z_s|x_{bow}, \hat{y})}{q(z_s|x, \hat{y})}] \\
& = EBLO_{x_{bow,\hat{y}}} - D_{KL}(p(z_s|x_{bow}, \hat{y})||q(z_s|x, \hat{y}))
\end{aligned}
$$

$$
\begin{aligned}
& ELBO_{x_{bow,\hat{y}}} \\
& = \mathbb{E}_{z_s}[\log p(x_{bow}, \hat{y}, z_s)] - \mathbb{E}_{z_s}[\log q(z_s|x, \hat{y})] \\
& = \mathbb{E}_{z_s}[\log p(x_{bow}|\hat{y}, z_s)] + \mathbb{E}_{z_s}[\log p(\hat{y}, z_s)] - \mathbb{E}_{z_s}[\log q(z_s)|x, \hat{y})] \\
& = \mathbb{E}_{z_s}[\log p(x_{bow}|\hat{y}, z_s)] + \mathbb{E}_{z_s}[\log p(\hat{y}|z_s)] + \mathbb{E}_{z_s}[p(z_s)] - \mathbb{E}_{z_s}[\log q(z_s)|x, \hat{y})] \\
& = \mathbb{E}_{z_s}[\log p(x_{bow}|\hat{y}, z_s)] + \mathbb{E}_{z_s}[\log p(\hat{y}|z_s)] - D_{KL}(q(z_s|x, \hat{y})||p(z_s))
\end{aligned}
$$

Where $p(z) = p(z_s) = \mathcal{N}(0, I)$ is a zero mean diagonal multivariate Gaussian prior, hence the $D_{KL}(q(z|x)||p(z))$ and $D_{KL}(q(z_s|x, \hat{y})||p(z_s))$ will be

$$
\begin{aligned}
& p(z) = p(z_s) = \mathcal{N}(0, I) \\
& D_{KL}(q(z|x)||p(z)) = 0.5(\sigma^2 + \mu^2 - \log(\sigma^2) - 1) \\
& D_{KL}(q(z_s|x, \hat{y})||p(z_s)) = 0.5(\sigma_s^2 + \mu_s^2 - \log(\sigma_s^2) - 1)
\end{aligned}
$$
