# Supplementary material for: Classification aware neural topic model for COVID-19 disinformation categorisation
Source: PLoS One. 2021 Feb 18;16(2):e0247086. doi: 10.1371/journal.pone.0247086 (PMC7891716; doi:10.1371/journal.pone.0247086)
Supplement: S6 Appendix — (PDF) [file pone.0247086.s006.pdf]

S6 Appendix – Extra Experimental Details

The bag-of-words pre-processing step is the same as [12]: All characters are transformed to lower case; stopwords[23], punctuation, all tokens less than 3 characters and all tokens that include numbers are removed.

The pre-processing step for the BERT representation is different from the bag-of-words pre-processing. For the COVID-19 corpus, all characters are lowercased, and tokenised by the BERT tokeniser from Huggingface[24] [17] Library.

The ADAM optimiser parameters are default from the Pytorch Library: Learning Rate = 0.001, betas=(0.9, 0.999). The number of training epochs are 200 as in [12], with early stopping when no training loss (classification loss for CANTM) decrease after 4 epochs.

The fine tuning layers for BERT (Huggingface BERT-base implementation) are:

- encoder.layer.11.attention.self.query.weight,
- encoder.layer.11.attention.self.query.bias,
- encoder.layer.11.attention.self.key.weight,
- encoder.layer.11.attention.self.key.bias,
- encoder.layer.11.attention.self.value.weight,
- encoder.layer.11.attention.self.value.bias,
- encoder.layer.11.attention.output.dense.weight,
- encoder.layer.11.attention.output.dense.bias,
- encoder.layer.11.intermediate.dense.weight,
- encoder.layer.11.intermediate.dense.bias,
- encoder.layer.11.output.dense.weight,
- encoder.layer.11.output.dense.bias

The number of parameters in CANTM (includes BERT) is 110,464,382 and number of trainable parameters is 8,066,942. The experimental hardware is: Intel(R) Xeon(R) Bronze 3204 CPU, TITAN RTX GPU, average epoch run time for COVID corpus is 41 seconds. The full list of number of parameters and epoch times is shown in S3 Table. Please note that Gensim LDA does not have GPU support, hence it running on a single core CPU.

[23] snowball.tartarus.org/algorithms/ english/stop.txt
[24] `https://github.com/huggingface/transformers`

Model	num. params	epoch time (sec.)
CANTM	110,464,382	41
BERTraw	109,489,930	36
BERT	109,521,200	37
SCHOLAR	740,360	0.05
NVDMb	109,661,140	37
NVDMo	1,152,600	20
LDA	151,750	0.6

S3 Table. Number of parameters and epoch training time. Gensim LDA does not have GPU support
