# Supplementary material for: Classification aware neural topic model for COVID-19 disinformation categorisation
Source: PLoS One. 2021 Feb 18;16(2):e0247086. doi: 10.1371/journal.pone.0247086 (PMC7891716; doi:10.1371/journal.pone.0247086)
Supplement: S7 Appendix — (PDF) [file pone.0247086.s007.pdf]

S7 Appendix - CANTM confusion matrix

Pred/True	PubAuth	CommSpread	MedAdv	PromActs	Consp	VirTrans	VirOrgn	PubPrep	Vacc	None
PubAuth	174	22	4	31	1	8	0	6	2	3
CommSpread	18	163	5	11	2	5	3	11	2	4
MedAdv	3	3	147	6	4	9	0	0	5	0
PromActs	38	17	0	149	5	3	0	5	3	1
Consp	11	10	1	10	45	4	12	1	2	1
VirTrans	7	14	11	1	4	33	7	0	3	0
VirOrgn	0	5	3	0	10	2	41	1	1	0
PubPrep	16	17	0	7	0	1	0	19	0	1
Vacc	1	0	14	3	1	3	1	0	52	1
None	7	20	1	5	2	2	1	3	1	1

S4 Table. CANTM confusion matrix
