# Supplementary material for: Classification aware neural topic model for COVID-19 disinformation categorisation
Source: PLoS One. 2021 Feb 18;16(2):e0247086. doi: 10.1371/journal.pone.0247086 (PMC7891716; doi:10.1371/journal.pone.0247086)
Supplement: S8 Appendix — (PDF) [file pone.0247086.s008.pdf]

S8 Appendix – Classification-Aware Topics Examples

Table S5 Table shows examples of classification-aware topics. Classification-aware topics are derived from R_s in M2 decoder (Section 3.1.5), hence R_s is 'aware' the pre-defined classes but not directly 'associate' with it.

Topics 1 and 4 are related to Public Authority about financial actions and official announcement. Topic 2 concerns a Conspiracy theory that is related to 'virus is lab created as war weapon'. Topic 3 is about economic influences from COVID-19 in Community Spread, and Topic 5 is related to Community Spread in South America.

Topic 1	cure warned kill notice current diseases attending human welfare suspended
Topic 2	demonstration nih dies kill nature human iraq someone war encourage
Topic 3	risk hindu san fall economic coronavirus conflict unit bars text
Topic 4	coronavirus prevent novel kill germany claim un gov eating document
Topic 5	ecuador first case buried end amazonas distributing recommended decreased april

S5 Table. COVID-19 classification-aware topics from unlabelled data
